# How the Protein Data Bank changed biology: An introduction to the JBC Reviews thematic series, part 1

**DOI:** 10.1016/j.jbc.2021.100608

**Published:** 2021-03-27

**Authors:** Helen M. Berman, Lila M. Gierasch

**Affiliations:** 1 Department of Chemistry and Chemical Biology, Rutgers, The State University of New Jersey, Piscataway, New Jersey, USA; 2 Department of Biological Sciences and Bridge Institute, University of Southern California, Los Angeles, California, USA; 3 Departments of Biochemistry & Molecular Biology and Chemistry, University of Massachusetts, Amherst, Massachusetts, USA

**Keywords:** crystal structure, cryo-EM, protein structure, nucleic acid structure, NMR, BNL, Brookhaven National Laboratory, PDB, Protein Data Bank, RCSB, Research Collaboratory for Structural Bioinformatics

## Abstract

This collection of articles celebrates the 50th anniversary of the Protein Data Bank (PDB), the single global digital archive of biological macromolecular structures. The impact of the PDB is immense; we have invited a number of top researchers in structural biology to illustrate its influence on an array of scientific fields. What emerges is a compelling picture of the synergism between the PDB and the explosive progress witnessed in many scientific areas. Availability of reliable, openly accessible, well-archived structural information has arguably had more impact on cell and molecular biology than even some of the enabling technologies such as PCR. We have seen the science move from a time when structural biologists contributed the lion’s share of the structures to the PDB and for discussion within their community to a time when any effort to achieve in-depth understanding of a biochemical or cell biological question demands an interdisciplinary approach built atop structural underpinnings.

A bit of history: In the 1960s when the very first protein structures began to be published, groups of scientists in the United States and Europe began to discuss the possibility of creating an archive for these data. Informal and formal meetings among interested parties were held, and one such meeting led to a petition directed at the crystallographic community to create a repository for crystallographic data accessible to all. The motivation behind the many discussions and the petition were many: Those who were determining the structures were being asked to share their data, which in those days was very challenging. Others began to sense that the data could yield some very interesting science and were keen to be able to analyze the structures. At the Cold Spring Harbor Symposium held in 1971, a group of scientists who had been involved in the earlier discussions (including one of us [H.B.]) approached Walter Hamilton, a prominent crystallographer at Brookhaven National Laboratory (BNL), and raised the possibility of a Protein Data Bank (PDB). He immediately said he would do it and promptly flew to England to initiate a collaboration with Olga Kennard, the head of the Cambridge Crystallographic Data Centre, to set up the PDB. An announcement of the fledgling archive appeared in October 1971 (1). Edgar Meyer, also at BNL, who developed protein visualization software, and Helen Berman began to work on the project with only a handful of structures. After Hamilton’s untimely death in 1973, Tom Koetzle, a postdoctoral fellow at BNL, took over the leadership and so the work continued.

At first, the PDB grew very slowly, and Koetzle put in significant effort to convince people to deposit their data. Ten years later, members of the community began to make very public demands that deposition be mandatory. After many discussions and yet another petition to the structural biology community—this one led by Fred Richards of Yale—guidelines were put in place by the International Union of Crystallography for deposition of coordinates as a condition of publication (2). More structures were deposited, at first mostly by crystallographers. As other methods for structure determination emerged, such as NMR spectroscopy and cryo-EM, those structures became a part of the PDB. In 1999, when there were 9000 structures in the PDB, the management was taken over by the Research Collaboratory for Structural Bioinformatics (RCSB), a consortium consisting of Rutgers, the San Diego Supercomputer Center, and the National Institute of Standards and Technology. In 2003, the Worldwide PDB was created to ensure that there would be a single global archive for structural data and that deposited data would be processed with uniform standards (3). The initial members were the RCSB PDB, Macromolecular Structure Database (MSD) later called Protein Data Bank Europe (PDBe), and Protein Data Bank Japan (PDBj). In 2006, the database created for NMR spectra called BioMagResBank (BMRB) joined.

The last 20 years has seen massive growth of the PDB, both in terms of the number of structures and complexity. Today, the archive contains more than 175,000 structures ranging in molecular weight from less than 20,000 Da to more than 380,000 Da. Complexes with more than 1000 components are now part of the PDB. The emphasis on quality control has grown, with expert task forces making recommendations for how best to validate structures determined by X-ray crystallography, NMR spectroscopy, and 3D electron microscopy. As integrative models are computed from data generated by several different methods, a special project is underway to create the necessary infrastructure to archive these structures.

These structures, standards, and projects are brought to life in this collection of JBC Reviews. We are particularly delighted to present this collection to you, as JBC has taken center stage in the history of the PDB for two key reasons: First, JBC was one of the first journals to require that authors deposit structural data reported in accepted articles to the PDB (4). This requirement is now widespread, if not ubiquitous among journals. Second, more structures now in the PDB have been published in JBC than in any other journal.

The structures in the PDB are diverse in almost every respect and cover multiple areas of biology and biochemistry. In this compendium, we have tried to cover at least part of the spectrum and give a sense of how much we have learned by being able to compare and study groups of structures. Many of the authors have chosen to describe the history of the field in terms of how the technology has evolved and in terms of the attitudes about structure sharing. In the paragraphs below, we offer previews of the review articles included in part 1 of this collection. Part 2 will include more reviews that celebrate additional scientific areas that have been profoundly touched by the creation of the PDB.

Many would agree that some of the most influential structures in the PDB are those of the ribosome, the complex factory made up of two large subunits, each with multiple chains of protein and RNA molecules, which produces proteins through the coordinated reactions of translation. **Peter Moore** (Yale University) provides in his article a historical account of the determination of the ribosome structure by four groups, three of whose leaders won Nobel Prizes in 2009 for their work (5). He points out the challenges that those first ribosome structures presented for the PDB. In retrospect, it is clear how fortunate it was that early RCSB PDB curators had been part of the Nucleic Acid Database project, enabling them to apply that experience and insight into representing these game-changing structures. Moore’s review captures the transition from reliance on X-ray crystallography to study these large machines to the current practice of using 3D electron microscopy for most ribosome structures, and why that is now the method of choice for these large assemblies. In addition to his description of the impactful work on the structure of the ribosome, Peter Moore adds unique reflections that his participation in structural biology from the “birth” of the PDB enables him to offer. He comments on the “state of play” in 1971 and the amazing group of scientists who presented at the Cold Spring Harbor Meeting described above. It is not surprising after reading his remarks that the creation of the PDB was one outcome of the meeting.

The importance of understanding the details of virus structure has been highlighted during this pandemic. In their review, **John ‘Jack’ Johnson** and **Art Olson** (The Scripps Research Institute) focus on icosahedral viruses, starting from the determination of the structure of two plant viruses in the 1980s followed by human viruses such as rhinovirus (6). Now there are hundreds of icosahedral virus structures in the PDB. They point out that, although these structures were of intense interest to the structural biology community, it was difficult to communicate the details to virologists. A graphics program, VIPER, provided that pathway and is also the basis for the way PDB represents these structures. The increasing use of 3D electron microscopy by crystallographers is described in one of **Johnson’s** publications (7). **Olson** also shares some of his early experiences in co-opting computer graphics programs to make the first movies of plant virus structures.

The discovery of the DNA structure using fiber diffraction data was the seminal event that paved the way to molecular and structural biology. Yet, it took almost two decades before an atomic level structure of a defined sequence was determined by Richard Dickerson’s laboratory at California Institute of Technology. **Stephen Neidle** (University College London School of Pharmacy) describes the history of DNA crystallography and how the earliest structures paved the way for understanding sequence-dependent structural diversity in oligonucleotides that are key to the recognition of DNA by proteins (8). The ability of G-rich tracts to form quadruplexes, far from being a biophysical artifact, underlies their role in human telomeres and allows them to assume a variety of functional topologies. And, more recently, deoxyribozymes that cleave RNA have been discovered and analyzed. Neidle highlights the important role that the PDB plays in ensuring the quality of the structures that are used for computational analyses and drug design.

The last 50 years has also seen an amazing evolution in RNA structure. **Eric Westhof** (University of Strasbourg) and the late **Neocles Leontis** (Bowling Green University) trace the history, which began with the structure determinations of tRNA and small RNA fragments as models for the double helix (9). The discovery of ribozymes opened the door to the RNA world, which continues to surprise us with its new folds and functions. The rich set of folding rules derived from these structures is discussed, as are modeling efforts based on these rules. The authors point out the value of the PDB in assembling and curating these structures and the key importance of validation. They also comment on the early role that the Nucleic Acid Database played as a testbed for the new formats that allow large macromolecular assemblies such as the ribosome to be properly archived in the PDB. Westhof also includes a tribute to Leontis, who was an RNA scholar and his longtime collaborator.

Many biological recognition and regulatory processes rely on the surface composition of proteins—the “face” they present to the world around them. In eukaryotic systems, carbohydrate modifications on the surfaces of proteins are extremely widespread. Yet, these decorations on glycoproteins proved elusive structurally for many years, in part because of heavy reliance on bacterial expression systems for preparation of adequate amounts of proteins for crystallography. In addition, the heterogeneity and dynamic character of carbohydrate modifications on proteins stymied crystallography for many years. **James Prestegard** (University of Georgia) reviews the breakthroughs that led to structural descriptions of the carbohydrate components of glycoproteins and how the resulting insights have elucidated biological puzzles (10). Central to this challenging area of structural biology was the deployment of NMR as a key method to determine the compositions and structures of the carbohydrate components of glycoproteins. The PDB has welcomed NMR structures for many years, and glycoproteins represented an example where synergistic use of multiple methods was essential to structural advances.

Membrane proteins have also presented unique challenges for structure determination because of the intimate dependence of their structural integrity on the anisotropic environment in which they function. **Robert Stroud** (University of California, San Francisco) and coworkers have beautifully described the massive progress that has occurred in the structural biology of membrane proteins, how breakthroughs were achieved by discovery of productive crystallization methods, and the growing number of examples now in the PDB that are leading to stunning advances in our understanding of many biological systems (11). This area of structural biology has been greatly facilitated by recent progress in cryo-EM.

Indeed, cryo-EM has emerged as one of the fastest growing methods for structure determination. **Wah Chiu** (Stanford University) and colleagues provide an historical overview of the key technical advances in sample preparation, instrumentation, and computer software that have contributed to the remarkable growth in the number of structures and vast improvements in resolution (12). He discusses the community activities that have led to the creation of data archives for maps and models, and the global collaboration that made it possible for both types of data to be deposited *via* the single Worldwide PDB deposition system. The development of validation criteria for maps and models has been facilitated by "Challenges" organized by the Electron Microscopy Data Resource (https://challenges.emdataresource.org/). The authors end their piece by highlighting the importance of validation reports in raising the quality of final structures in the PDB.

Computational biology has made major leaps over the lifetime of the PDB, in no small measure because of the rich data available in solved protein structures. **Tanja Kortemme** (University of California, San Francisco) and her colleague Xingjie Pan describe how the field of protein design has been enabled by access to the extensive structural data in the PDB (13). The ability to use the principles that Nature illustrates in evolutionarily honed structures to build up guiding principles for designed proteins has been crucial. As beautifully presented in this JBC Review, we are seeing the field of computational protein design achieve landmark goals: design of novel proteins with desired functions, design of folds never previously observed, and mimicking the impact of evolution on naturally occurring protein families in families of designed proteins. Not only do these advances open many doors for engineering novel proteins but also they inform the field of structure prediction, as strikingly illustrated by recent artificial intelligence prediction methods (14).

The advances in computational biology have also fed impressive developments in methods to exploit the breadth of available structural data, and **Barry Honig** (Columbia University) and colleagues describe how one new area, computational systems biology, has emerged as a way to tackle complex functional relationships among proteins (15). They illustrate how computational methods can lead to structure-informed relationships between proteins. These in turn can reveal protein interactomes and provide testable models for genetic screens. In the long run, such methods may lead to novel therapeutic strategies based on linking protein structure space with chemical compound space.

Knowledge of protein structures is essential to our understanding of health and disease and for drug discovery. In his review, **Stephen K. Burley** (Rutgers University) discusses how structures are used in many stages of drug development: target validation, druggability, small-molecule binding to drug targets, structure-guided lead optimization, and optimization of pharmacokinetic properties (16). He describes quantitative analyses of the impact of PDB structures on the drug approval process, and three case studies for how structure-guided approaches were key in the approvals of small-molecule antineoplastic drugs are provided as illustration.

This splendid array of scholarly and forward-looking JBC Reviews are a compelling testament to the power of openly accessible data depositories, with the amazing biological discoveries fueled by structural information available in the PDB serving as a premier example. We hope you join us in saluting the structural biology community for its prescience in establishing the PDB a half a century ago and in basking in the beauty and fundamental knowledge structural information has brought to biology. There is more to come in this celebratory collection: stay tuned for part 2!

## Conflict of interest

The authors declare that they have no conflicts of interest with the contents of this article.
